# Does the intermittent Pringle maneuver affect the recurrence following surgical resection for hepatocellular carcinoma? A systematic review

**DOI:** 10.1371/journal.pone.0229870

**Published:** 2020-03-11

**Authors:** Nanping Lin, Jingrong Li, Qiao Ke, Fuli Xin, Yongyi Zeng, Lei Wang, Jingfeng Liu

**Affiliations:** 1 Department of Hepatopancreatobiliary Surgery, Mengchao Hepatobiliary Hospital of Fujian Medical University, Fuzhou, Fujian, China; 2 The First Affiliated Hospital of Fujian Medical University, Fuzhou, Fujian, China; 3 Department of Laboratory, Fujian Medical University Union Hospital, Fuzhou, Fujian, China; 4 Department of Radiation Oncology, Mengchao Hepatobiliary Hospital of Fujian Medical University, Fuzhou, Fujian, China; Cincinnati Children’s Hospital Medical Center, UNITED STATES

## Abstract

**Background and aim:**

To evaluate the effect of intermittent pringle maneuver (IPM) on the long-term prognosis and recurrence of hepatocellular carcinoma (HCC).

**Methods:**

Eligible studies were identified by PubMed and other databases from Jan 1st 1990 to Mar 31st 2019. Hazard ratios (HR) with 95% confidence interval (CI) were calculated to evaluate the effects of IPM on the long-term prognosis and recurrence of patients with HCC.

**Results:**

Six studies were enrolled in this meta-analysis. Results showed that there were no differences between IPM group and non-IPM group in the pooled HRs for the overall survival (OS) and disease-free survival (DFS) (HR 1.04, 95%CI 0.84~1.28, P = 0.74; HR 0.93, 95%CI 0.81~1.07, P = 0.29; respectively). However, subgroup analysis showed that the pooled Odd ratios (OR) for the 1-year OS and DFS rates of the IPM group when compared with the non-IPM group were 0.65 (95% CI 0.45~0.94, P = 0.02), 0.38 (95% CI 0.20~0.72, P = 0.003), respectively. In addition, there were no significant differences in the proportions of liver cirrhosis, HBsAg (+), Child-Pugh A class, multiple tumor, vascular invasion, and major hepatectomy between groups of IPM and non-IPM.

**Conclusion:**

Since IPM would increase the risk of early-recurrence, it should be used cautiously in the procedure of hepatectomy for resectable HCC. However, the current conclusion needs further validation.

**Trial registry number:**

CRD 42019124923

## Introduction

The incidence of hepatocellular carcinoma (HCC) is increasing stably worldwide[1,2], although it is decreasing in eastern Asia countries, especially in China[3]. Liver transplantation and ablation techniques have been progressing remarkably in recent years[1,4,5], liver resection still remains the most preferred kind of strategy for HCC. However, the incidence of recurrence following resection, especially the early recurrence, is still high[6,7,8].

Ischemia-reperfusion (I/R) injury caused by blood occlusion might contribute to the recurrence of HCC, and potential mechanisms were as following: 1) upregulation of vascular endothelial growth factor[9], 2) activation of hepatic stellate cells[10], 3) promotion of cell signaling associated with tumor cell adhesion, invasion, and migration[11], 4) delayed damage to the remnant liver [12,13]. Intermittent pringle maneuver (IPM) is the most common kind of blood occlusion worldwide, mainly because it would reduce the risk of I/R when compared with PM[14,15], which is confirmed in the animal model [16]. However, worries about recurrence correlated with IPM never lessens[17,18].

Relevant clinical trials evaluating the impact of IPM on the long-term prognosis after curative resection have been rarely published openly in the previous decades, and the currently identified studies are almost come from eastern countries[19,20,21]. Until recently, a study from a western series reported that IPM did not increase the risk of recurrence and decrease long-term survival[22]. Considering that most of the current studies are retrospective studies, we wanted to conduct a systematic review and observe whether IPM could affect the prognosis and recurrence of HCC.

## Methods

This systematic review was performed according to the guidelines of the Preferred Reporting Items for Systematic Reviews and Meta-Analyses (PRISMA) and Assessing the Methodological Quality of Systematic Reviews (AMSTAR).

### Literature search

A comprehensive search was conducted by two independent researchers to identify all the eligible studies evaluating the clinical value of IPM for HCC. English electronic databases such as PubMed, MedLine, the Cochrane Library, Web of Science, EMbase were used to seek the literature, from Jan 1st 1990 to Mar 31st 2019. Following terms and strategy were used to seek the eligible studies: (“hepatocellular carcinoma” or “HCC”) AND (“liver resection” or “hepatectomy” or “surgical resection” or “resection”) AND (“blood occlusion” or “hepatic blood occlusion” or “intermittent Pringle maneuver” or “intermittent Pringle manoeuvre” or “IPM”). Furthermore, any potentially eligible studies were identified manually from the included studies, reviews, letters, and comments. Of note, only studies written in English, either retrospective or prospective, were enrolled.

### Selection criteria

Inclusion criteria: 1) patients defined only as HCC; 2) IPM performed in the surgery; 3) no hepatic blood occlusion as the control group; 4) outcomes including long-term, such as disease–free survival (DFS) and overall survival (OS); 5) studies either retrospective or prospective.

Exclusion criteria: 1) liver cancers including intrahepatic cholangiocarcinoma, and metastatic liver cancer; 2) hepatectomy designed not for HCC, such as hepatic hemangioma, hepatolithiasis, and so on; 3) continue PM or selective PM; 4) case reports, letters, reviews and conference reports; 5) studies based on overlapping cohorts derived from the same center; 6) data unavailable.

In case of results reported from the same center more than once, the latest was extracted.

### Data extraction

Data was extracted including all of the following: ① general data, such as title, first author, publication data and literature source, and so on; ② baseline characteristics, such as sex, age, liver cirrhosis, HBsAg, liver function, tumor number, surgical techniques, occlusion time, and vascular invasion and so on; ③ primary endpoint, OS and DFS. Liver function was evaluated by Child-Pugh grading, multiple tumors were defined as tumor number ≥2, surgical techniques included minor or major resection, and vascular invasion was defined as tumors invaded into macro or micro-vascular.

All data were extracted and assessed by two independent investigators with predefined forms such as baseline characteristics and outcomes from each study. In the case of disagreement, a third investigator intervened for a conclusion. Hazard ratios (HRs) and its 95% confidence intervention (CI) were extracted from original studies, or calculated by Engauge Digitizer 4.1 according to Kaplan-Meier curve[23,24].

### Intervention & outcome definition

Hepatectomy, regardless of minor or major, was usually performed using transactional approach of the finger combined with cavitron ultrasonic surgical aspirator (CUSA) or harmonic apparatus or Peam clamp fracture. Minor hepatectomy was defined as resection of less than three segments, while major resection was as three or more.

IPM was carried out using the tightening of a rubber tube encircling the hepatoduodenal ligament. The procedure was usually clamping within 15 minutes of ischemia followed by 5 minutes of reperfusion, but it varied a little among different centers. Continues PM was defined as PM continued in the procedure of hepatectomy without any reperfusion. Selective PM was defined as only left or right hepatic inflow, or selected hepatic inflow supplied the tumor was blocked in the procedure of hepatectomy.

OS was defined as the time (in months) from hepatectomy to death, and the data were censored at the date of the latest follow-up in the absence of death. DFS was defined as the time (in months) from hepatectomy to recurrence, and the data were censored at the date of the latest follow-up in the absence of recurrence.

### Quality assessment

The quality of the included studies was assessed by the modified Newcastle-Ottawa Scale (NOS) [25]for case control studies based on the three main elements: the selection of study groups (0–4 points), the comparability between the two groups (0–2 points), and the determination of either the exposure or the outcome of interest (0–3 points). A full score was 9, and studies scored above 5 were considered to be of high quality.

### Statistical analysis

The systematic review and meta-analysis were registered at http://www.researchregistry.com and performed using RevMan Version 5.3 and Stata 14. The χ^2^ test and *I*^*2*^ statistics were used to assess the heterogeneity; *P*<0.10 or *I*^*2*^>50% were considered as significant heterogeneity. When the hypothesis of homogeneity was not rejected, the fixed-effects model was used to estimate the case with homogeneity, and the random-effects model was used for the cases with significant heterogeneity. Hazard ratios (HR)was evaluated for the DFS and OS, and Odd ratios (ORs) were for 1-, 3, 5-year survival rates and DFS rates, as well as clinical/pathological characteristics, accompanied with 95% confidence intervals (CI). A sensitivity analysis was performed as follows: one study at a time was removed and the others analyzed to estimate whether the results could have been affected markedly by single study[26].

## Results

### Base characteristic of the included studies

**Fig 1:**
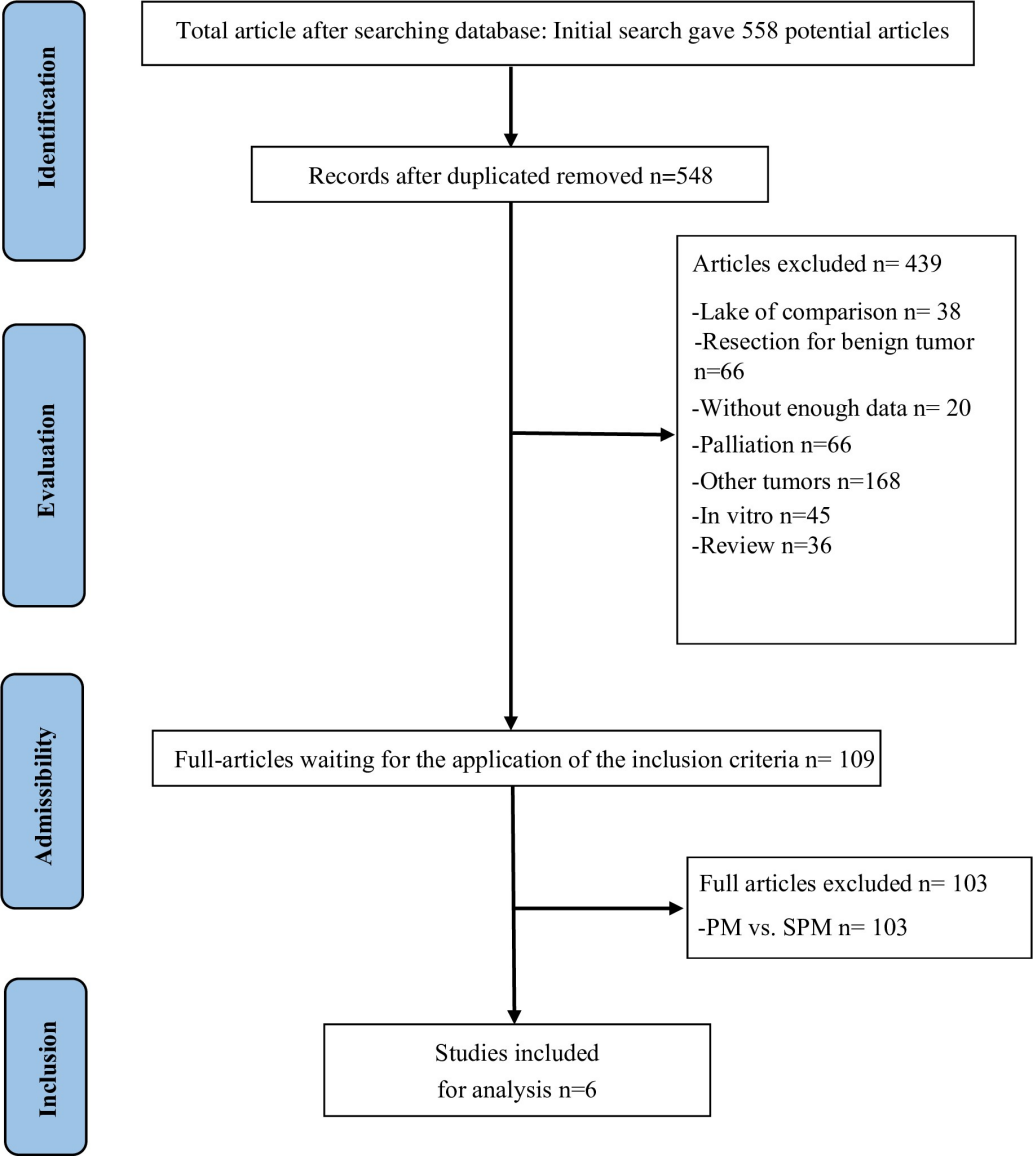
Flowchart of study selection process for meta-analysis.

Initially, 558 reports were identified by two independent reviewers, and then 10 articles were excluded after duplicate removal by NoteExpress 3.1. After browsing titles and abstracts, 439 records were excluded, including 38 were for lack of comparison, 66 were for benign disease, 20 were for data unavailable, 66 were for palliative treatment, 168 were for not specified for HCC, 45 were for in vitro studies, 36 were for reviews. Among the remained 109 records, 103 records were excluded for comparison PM with selective PM, Finally, six researches[21,22,27,28,29,30] were included for analysis. Details were depicted specifically in Fig 1.

In total, 5275 patients were enrolled in this meta-analysis, with 3290 cases in the IPM group and 1985 cases in the non-IPM group. The characteristic and quality of the included trials were shown in Table 1. However, only one RCT[29] was eligible. The scores ranged from 7 to 8, indicating that all the studies were of high quality (Table 1). Of note, five of the six included studies came from China[21,27,28,29,30].

**Table 1:** Basic characteristics of the trials included.

Study	Country	Study year	Design of studies	Follow-up (months)	IPM				Non-IPM			NOS
					NO.	Age (Year)	Sex F:M	occlusion time (min)	NO.	Age (Year)	Sex F:M	
Xia 2013	China	2001–2006	RCS	120	224	48 (21–78)	51:173	50 (30–98)	162	57 (18–79)-	43:119	8
Huang 2014	China	1998–2008	RCS	60	931	56.4±17.2	264:667	48.7±37.6-	618	54.2±22.1	145:473-	7
Hao 2016	China	2010–2012	RCS	25	206	52.9	45:161	29.1±9.8-	60	55.0	12:48-	8
Hao 2017	China	2007–2010	RCS	60	113	51.7	37:76	-	52	55.0	15:37	6
Famularo 2017	Italy	2001–2015	RCS	60	176	65.1 (58.2–72)	31:145	23 (14–30)	265	67.6 (59.2–73.9)	66:199-	8
Lee 2018	China	2013–2016	RCT	48	50	59.5 (38.0–84.0)	7:43-	45.0 (15.0–60.0)	50	62.0 (27.0–78.0)	11:39	8

*IPM: intermittent Pringle Maneuver; F:M, female: male; RCS: retrospective cohort study; RCT: randomized controlled trial; NOS, Newcastle-Ottawa Scale

### Meta-analysis of clinical and pathological characteristics related to prognosis

Baseline characteristics related to prognosis, including liver cirrhosis, status of HBsAg, liver function, tumor number, vascular invasion, and surgical techniques, were analyzed to evaluate the potential bias resulted from cofounding factors. Results showed that there were no significant differences in the proportions of liver cirrhosis, HBsAg (+), Child-Pugh A class, multiple tumors, vascular invasion, and major hepatectomy between the IPM group and non-IPM group. The pooled ORs for each potential risk factor were depicted specifically in Table 2.

**Table 2:** Clinical and pathological characteristics of the trials included.

Factor Study	Liver cirrhosis		HBsAg (+)		Child type A		Multiple tumor		Major hepatectomy		Vascular invasion	
	IPM	Non-IPM	IPM	Non-IPM	IPM	Non-IPM	IPM	Non-IPM	IPM	Non-IPM	IPM	Non-IPM
Xia 2013	169	128	209	149	141	101	79	40	93	77	76	65
Huang 2014	682	322	717	469	-	-	283	185	416	289	-	-
Hao 2016	-	-	163	50	130	40	205	101	127	35	-	-
Hao 2017	-	-	-	-	74	35	76	21	73	27	-	-
Famularo 2017	144	214	-	-	160	248	42	54	22	36	-	-
Lee 2018	28	25	35	40	50	50	13	12	15	16	14	17
***I***^**2**^ **(P value)**	92%(P<0.01)		0%(P = 0.67)		0%(P = 0.81)		47%(P = 0.08)		63%(P = 0.01)		0%(P = 0.98)	
**OR (95% *CI*)**	1.19(0.66,2.15)		1.02(0.87,1.21)		0.91(0.68,1.21)		1.22(0.98,1.52)		1.10(0.84,1.44)		0.76(0.52, 1.11)	

*IPM: intermittent Pringle Maneuver; OR: odd ratio; CI: confident index.

### Primary endpoints

**Fig 2:**
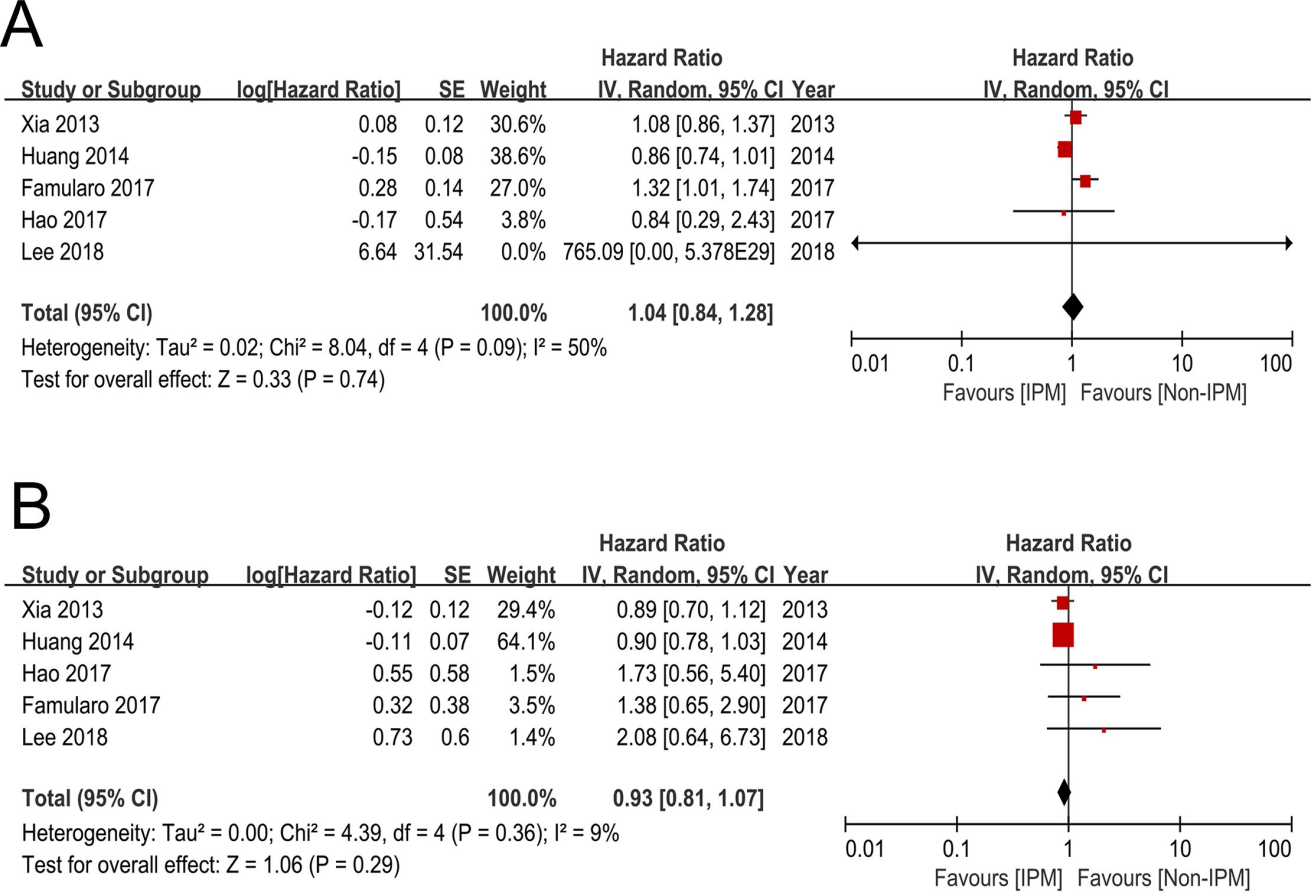
Forest plots of OS and DFS rate comparing IPM and non-IPM.

OS was evaluated in five studies[21,22,27,28,29], and significant heterogeneity was observed among included studies (*I*^2^ = 50%, P = 0.09). The pooled HR was determined by random-effect model, and results showed that there were no significant differences between IPM group and non-IPM group (HR 1.04, 95%CI 0.84~1.28, P = 0.74; Fig 2A). A sensitivity analysis was performed, and the result was not affected by any single study.

DFS was evaluated in the same five studies[21,22,27,28,29], but no significant heterogeneity was observed among included studies (*I*^2^ = 9%, P = 0.36). The pooled HR was determined by fixed-effect model, and results showed that there were no significant differences between IPM group and non-IPM group (HR 0.93, 95%CI 0.81~1.07, P = 0.29; Fig 2B). Similarly, the result was not affected by any single study.

### Subgroup analysis

**Fig 3:**
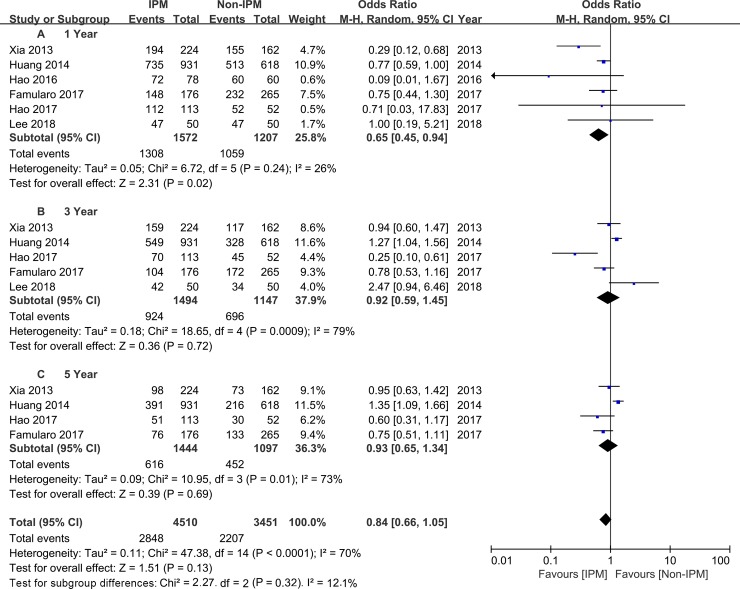
Subgroup analysis of 1-, 3-, and 5-year survival rates comparing IPM and non-IPM.

**Fig 4:**
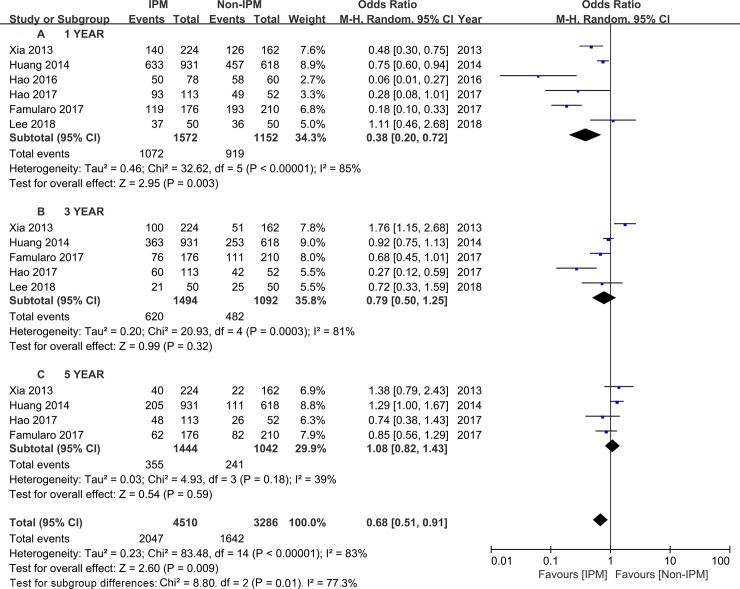
Subgroup analysis of 1-, 3-, and 5-year DFS rate comparing IPM and non-IPM.

The 1-, 3-, and 5-year survival rates were evaluated in six[21,22,27,28,29,30], five[21,22,27,28,29], and four[21,22,27,28] studies, respectively. A random-effect model was used, and the pooled ORs for the 1-, 3-, and 5-year survival rates of the IPM group when compared with the non-IPM group were 0.65 (95% CI 0.45~0.94, P = 0.02; Fig 3A), 0.92(95% CI 0.59~1.45, P = 0.72; Fig 3B), and 0.93 (95% CI 0.65~1.34, P = 0.69; Fig 3C), respectively. Similar results were observed in the pooled ORs for DFS (1-year DFS: OR = 0.38, 95% CI 0.20~0.72, P = 0.003, Fig 4A; 3-year DFS: OR = 0.79, 95% CI 0.50~1.25, P = 0.58; 5-year DFS: OR = 1.08, 95% CI 0.82~1.43, P = 0.59; respectively).

## Discussion

Concerns on recurrence resulted from IPM have always been hovering in the heads of hepatobiliary surgeons[17,18]. This was the first systematic review, which was designed to evaluate the effect of IPM on the long-term outcomes and recurrence. A total of 5275 patients were included in this study. Result confirmed that clinical and pathological characteristics including the proportions of liver cirrhosis, HBsAg (+), Child-Pugh A class, multiple tumors, vascular invasion and major hepatectomy were comparable between groups of IPM group and non-IPM. IPM was found not to affect the long-term OS and DFS, but it was associated with decreased 1-year survival rate and DFS rate. Hence, we concluded that IPM would not influence the long-term prognosis of patients, but it would affect the short-term outcomes.

IPM, as a modified model of PM, has been performed prevalently worldwide. IPM is comparable with PM in the controlment of intraoperative blood loss and prevention of postoperative liver dysfunction[31,32], but as for long-term efficacy it remains controversial. OS and DFS are reported to be irrelevant with IPM for colorectal liver metastasis [33,34], but it has yet to be known for HCC. In this study, six studies[21,22,27,28,29,30] were eligible for this meta-analysis, including the latest report from the western series[22]. Resulted showed that long-term outcomes were comparable between groups of IPM and non-IPM, which was in line with the most of the eastern series and the newly-published western series.

Early recurrence occurring within 1~2 years, is an important independent risk factor for poorer long-term outcome of HCC patients[35]. I/R caused by blood occlusion is one of the important mechanisms for tumor recurrence[36], which was confirmed repeatedly in vitro and in animal models. The detailed mechanisms are as follows: 1) aggressive characteristics of tumor, such as invasiveness, adhesion, and transitivity were boosted by I/R[37,38]; 2) both inflammatory factors and cytokines factors correlated with tumor recurrence were up-regulated by I/R injury[38]; 3) endotoxin-mediated Toll-Like Receptor 4 (TLR-4) was engaged by mesenteric congestion related to PM[39]. In this study, the pooled OR for the 1-year survival rate and DFS rate were much lower in the IPM group than those in the non-PM group (OR 0.65, 95% CI 0.45~0.94, P = 0.02; OR 0.38, 95% CI 0.20~0.72, P = 0.003; respectively). Hence, we concluded that IPM might be associated with early recurrence of HCC and poorer short-term prognosis.

The detailed procedure of IPM is slightly different from each center, with repeated clamping less than 10~20 min followed by 5~10 min of reperfusion[22,29,40]. Prolonged PM duration was reported to be well tolerated for up to 2 hours[41], but the longer ischemia, the more severe reperfusion injury. Occlusion time and courses are typically correlated with the incidence of intraoperative blood loss, the rate of liver dysfunction of HCC[19,22,28,30], but it remains controversial whether they affect the long-term outcomes. Liu et al found that longer occlusion time increased the risk of early recurrence or shortened overall survival time, while a shorter duration decreased the risk of recurrence. However, subgroup analysis stratified by the occlusion time and course were not conducted in this study mainly because the most of the relevant data was unavailable. Among the included studies, the identified threshold effect of IPM on HCC recurrence was 60 min[42], but it remained unknown for 15min[19,22], 30 min[22] and 45 min[28,30]. Hence, studies focusing on the total ischemia time and the courses of occlusion are badly warranted.

In real world, HCC patients who had more sever cirrhosis, larger tumors, multiple tumors and poorer differentiated tumors were assumed to be arranged in the group of IPM group[22], indicating that the current results were often less convicting. However, prospective studies on this issue are generally hard to be carried out, and to the best of our knowledge, most of the previously registered trials have not yet been published openly up to now. In this meta-analysis, only one prospective study was identified. Hence, relevant analysis was conducted to decrease the influence of potential confounding factors related to the long-term outcomes. And results showed that the pooled OR for the proportions of liver cirrhosis, HBsAg (+), Child-Pugh A class, multiple tumor, vascular invasion and major hepatectomy were comparable between groups of IPM and non-PM, which indicated that the conclusion in this study was considerably convictive.

However, there were several restrictions in this meta-analysis. First, five of the six included studies were retrospective, which indicated that selection bias and recalling bias were hard to avoid. Second, only one western series[22] was identified in this meta-analysis, which would weaken the conclusion of this study because the epidemiology between the west and east was different. Third, the procedure of IPM was similarly worldwide, but the durations of each IPM were from 10 min to 20 min[22,29,40]. Fourth, occlusion time and course of IPM were also the key for I/R, but details on these issues were unavailable. Finally, confounding factors were inevitable and such corresponding subgroup analyses were unable to conduct, although baseline characteristics related to prognosis and recurrence were confirmed to be comparable between the two groups.

With the current data, IPM should be used cautiously in the procedure of hepatectomy for resectable HCC, since it would increase the risk of early-recurrence. However, more prospective multicenter trials are needed to furtherly verify this conclusion.

## Supporting information

S1 PRISMA checklist (DOC)
